# Feline oral squamous cell carcinoma and *Felis catus* papillomavirus: is it time to walk the path of human oncology?

**DOI:** 10.3389/fvets.2023.1148673

**Published:** 2023-05-17

**Authors:** Gennaro Altamura, Giuseppe Borzacchiello

**Affiliations:** Department of Veterinary Medicine and Animal Productions, University of Naples Federico II, Naples, Italy

**Keywords:** papillomavirus, *Felis catus*, oral squamous cell carcinoma, head and neck cancer, comparative oncology

## 1. Introduction

In humans, head and neck squamous cell carcinoma (HNSCC) is among the most common cancers worldwide. This severe disease affects the aerodigestive tract, including oral cavity and oropharynx (1). Tobacco smoking, alcohol abuse and poor oral hygiene are believed to be the main risk factors. However, it is estimated that a subgroup of oral squamous cell carcinoma (OSCC), accounting for 25% of all HNSCCs, is associated with alpha high-risk human papillomavirus (HR-HPV) infection, with the HPV-16/-18 being the major responsible for cancer development. This particular type of tumor arises mainly at oropharyngeal sites, where persistent infection plays a key role toward neoplastic transformation. Viral oncogenes E6 and E7 drive carcinogenesis in infected cells by impairing the molecular pathways of two key tumor suppressors such as p53 and pRb (1). In veterinary oncology, association of PVs with SCC of the upper digestive tract has been ascertained in bovine and equine species (2–5). In cattle, bovine PV type-4 (BPV-4) is considered a main player in early steps of tumorigenesis leading to development of SCC of the esophagus, mouth, and oropharynx, along with environmental co-factors (2, 6). Furthermore, there is increasing evidence that *Equus caballus* PV-2, likely to be a co-causative agent of genital SCC in horses, is also involved in development of a subset of equine HNSCC, thus being considered an equine equivalent of HPV-16 (3–5). Studies in dogs have suggested a possible role of canine PVs in oral carcinogenesis, particularly in the transformation of oral papillomas into SCC (7–9). Similarly, a rising number of published work indicates that *Felis catus* papillomaviruses (*Fca*PVs) exhibit mucosal tropism, being consistently detectable in a subset of OSCC of cat and playing a co-causative role in the development of these tumors (10–16).

## 2. Association of FOSCC with *Fca*PVs infection

The first hints of a biologically significant association of feline OSCC (FOSCC) with *Fca*PVs came from the pioneering studies aimed at characterizing transcriptional activity of *Fca*PV type 2 (*Fca*PV-2) *in vivo* and its biological properties in feline living cells (10, 17). Here, viral DNA and gene expression were reported in one FOSCC case and *Fca*PV-2 E6 and E7 oncogenes appeared to exert transforming properties comparable to those of HR-HPVs associated with human HNSCC (10, 17). Additional clues were pointed out when *Fca*PV-2 was shown to be detectable and transcriptionally active in cell lines derived from cat gingival and tongue SCC (18). Moreover, these cell lines showed a molecular scenario compatible with a *Fca*PV-2 E6-dependent p53 degradation with similarities to that reported for HPV-16 E6 (18). Stronger evidence came from two later independent studies that reported detection of *Fca*PV-2 in a relevant subset of FOSCC from different geographical areas, with a prevalence of 31% (10/32) in Italy and ~58% (11/19) in Japan (12, 19). Importantly, the study conducted in Italy pointed out expression of *Fca*PV-2 oncogenes E6E7 in tumors and higher viral load compared to non-neoplastic oral mucosa harboring viral DNA, suggesting active infection and oncogenic functions (19). A later work from Germany confirmed expression of PV antigen in ~21% (5/21) of FOSCC and the presence of PV-induced cellular changes (koilocytes and inclusion bodies) in a subset of samples (15). Furthermore, a recent multicentric study demonstrated the presence of at least one *Fca*PV type (among these *Fca*PV-1/-2/-3/-4/-5) in ~21% (22/103) of a pool of FOSCC samples from Italy and Austria (11). Consistent data were presented in two independent published works conducted in USA and New Zealand, denoting the presence and high viral load of *Fca*PV-3 in 5% (1/20) of FOSCC but not in normal mucosa, and typical PV-induced cellular changes in a *Fca*PV-3 positive tumor sample (13, 14). Taking these studies together, the association rate of *Fca*PVs with FOSCC seems to fluctuate between 5 and 58%, however a conference paper from USA dated in 2015 reports that it can even reach 100% (12/12) (20).

In summary, the evidence of a co-causative role of *Fca*PVs in the development of FOSCC is as follows:

1) Different *Fca*PV types (-1/-2/-3/-4/-5) exhibit mucosal tropism (16, 21).
2) A subset of FOSCC samples is associated with *Fca*PVs DNA (11–14, 19, 20).
3) Detection of *Fca*PVs DNA in a subset of FOSCC is a common finding in different geographical areas, as per independent studies by different research groups (11–14, 19, 20).
4) There is histological, molecular and immunohistochemical evidence of PV active infection in a subset of FOSCC by different research groups (13, 15, 19).
5) *Fca*PV-2 displays high viral load and expression of E6E7 oncogenes in FOSCC samples (10, 19).
6) *Fca*PV-2 DNA is detectable and viral oncogenes are expressed in FOSCC-derived cell lines (18).
7) *Fca*PV-2 E6 and E7 oncoproteins exert transforming properties by impairing p53 and pRb pathways in feline living cells (10, 18, 22).
8) *Fca*PV-3 induces cellular changes compatible with PV-induced cancer and displays high viral load in FOSCC (13, 14).

Finally, as brilliantly summarized in a recent, excellent critical review of the literature, *Fca*PVs infection clearly emerges as a risk factor for a subset of FOSCC (~16%). Interestingly, the authors even warn that the number of PV positive cases might be underestimated, due to: (I) DNA fragmentation occurring in formalin fixed-paraffin embedded samples causing false negative PCR results. (II) The use of consensus primers, which exert lower sensitivity than type-specific primers. (III) Infection by genotypes not detectable by the primers employed in elder studies. (IV) The possible occurrence of the “hit and run” mechanism by which the virus may initially induce cellular transformation, to then disappear and go no longer detectable (23).

## 3. Discussion

Among the numerous diseases classified as HNSCC (SCC of oral cavity, oropharynx, nasopharynx, larynx and upper esophagus), HPV-positive SCC shows different biological features, genetic background and molecular markers compared to HPV-negative counterpart and is now considered as a distinct clinical entity (1, 24). Indeed, due to a less aggressive behavior combined with an improved response to therapies, the 3-year overall survival of patients bearing HPV-positive cancer is 82 vs. 57% of those affected by HPV-negative tumors (1, 24). Recent studies carried out in the context of global scale meta-analyses conducted by the International Agency for Research on Cancer (IARC) confirm improved survival in HPV positive patients, with oropharyngeal tumors driving this trend (25). These data further strengthen the rationale of numerous past, ongoing, and future studies aiming at de-intensificating therapeutic protocols against HPV-related OSCC (25, 26). The focus of these studies is to maintain high cure efficiency, reduce treatment-related toxicity, and preserve the quality of life at the same time (25, 26).

Therefore, HPV testing is a crucial step working as a link between the pathologist and the clinical oncologist toward a complete diagnostic, therapeutic and prognostic evaluation in the practice of cases of human OSCC. Immunohistochemistry (IHC) for p16 is considered a reliable test that serves as a surrogate for HPV detection, since it is a downstream effector of impaired pRb (24, 27). In doubtful cases, IHC may be further integrated by molecular tools such as *in situ* hybridization (ISH) for viral DNA and E6/E7 gene expression analysis (27).

**Figure 1:**
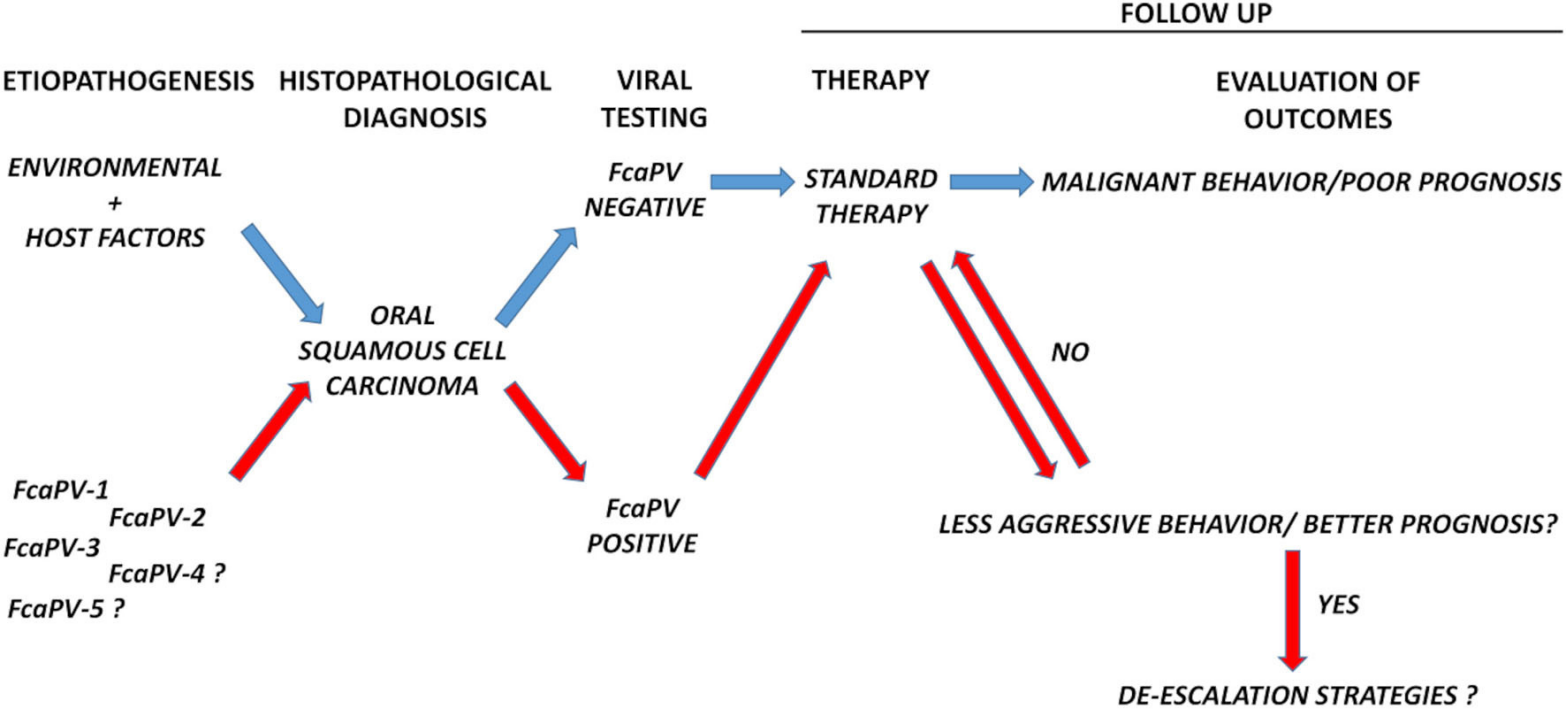
Schematic diagram showing the diagnostic workflow proposed in this article. Follow up of biological behavior and response to therapies of *Fca*PV-positive vs. *Fca*PV-negative tumors would help to understand whether, as in humans, the former constitutes a different oncological entity compared to the latter and possibly pave the way to de-escalation strategies. Viral testing would be a necessary, preliminary step before clinical handling.

Oral tumors are frequent in cats, among these SCC is the most common malignancy (28). Surgery, chemotherapy and radiation therapy are available as treatment options, however the prognosis is poor in most of the cases, leading to death or euthanasia (28). We still do not know whether *Fca*PV-related FOSCC may actually constitute a distinct oncological entity in terms of biological behavior as in human counterpart. There is a need to collect additional data; therefore, we ask ourselves and the broadest community of veterinary oncologists whether it is time to set-up large-scale strategies toward this goal by coordinating scientific efforts worldwide. This means to design multicentric follow up studies with the aim of monitoring the biological behavior and the evolution of these tumors over time, in order to understand whether they represent or not a different clinical subject compared to PV-negative disease (Figure 1). Evaluating the incidence of PV-related cancer in different oral sites would be of great interest as well, to see whether it preferentially develops at oropharynx also in felines. If so, this FOSCC subtype will further confirm its reliability as animal model of HPV-driven HNSCC and *vice versa* (29). Standard treatment for human HNSCC is surgery followed by high-dose cisplatin with adjuvant radiotherapy and it is widely ascertained that this protocol works particularly well for HPV positive tumors. Importantly, in IARC studies, surgery has been proven to be the most impactful treatment and further indications have emerged that chemo-radiotherapy might be de-escalated (25). Will a similar scenario be confirmed in feline species, it will be even more important to devise possible de-escalation strategies for *Fca*PV-positive patients (Figure 1). This would be achieved by setting up studies where modulating different combinations of surgery, chemo-, radio- and biological therapy to then compare primary (e.g., overall survival, disease-free survival) and secondary outcomes (e.g., loco-regional control) in differently treated experimental groups. Among the de-intensification strategies eligible for the treatment of human HNSCC, biological therapy based on the use of monoclonal antibody Cetuximab as adjuvant has been object of great attention (30). Hence, it is worth mentioning that our recent work in pre-clinical models of FOSCC has provided promising results, thus encouraging future studies in the feline counterpart (31). We imagine a future perspective where de-escalated therapies may provide advantages for cats, with a more favorable balance between therapeutic efficacy and welfare, and owners, in handling and economic terms. In such a context, viral testing would emerge as a necessary, preliminary step prior to the clinical management, as determined by the diagnostic algorithm in human practice. In this regard, a proportion of *Fca*PV-2 positive FOSCCs display p16 immunostaining, and this appears as a positive prognostic parameter in cats too. However, the role of p16 as marker of PV infection in this species is controversial (19, 23, 32), thus molecular tests (PCR and RT-PCR) would be recommendable (10, 19). Moreover, ISH would find possible application, considering that it is successfully employed for detection of *Fca*PVs DNA and mRNA in feline cutaneous SCC (33–35).

In conclusion, pending definitive data in humans, we believe it is time to begin studies in domestic feline, with the aim of studying the biology, clinical behavior and response to therapies of *Fca*PV-related FOSCC. In a future perspective, this would help to ameliorate the approach of the veterinary oncologists in terms of diagnosis, therapeutic and prognostic evaluation toward feline patients.

We are used to think of comparative oncology as the discipline in which studies on animal models pave new ways in human medicine. However, this would be the case were the “human model” traces the path for feline oncology, although we do not yet know what lies at the end of the road.

## Author contributions

GA and GB drafted the manuscript. All authors contributed to the article and approved the submitted version.
